# Effects of Chronic Stress from High Stocking Density in Mariculture: Evaluations of Growth Performance and Lipid Metabolism of Rainbow Trout (*Oncorhychus mykiss*)

**DOI:** 10.3390/biology13040263

**Published:** 2024-04-16

**Authors:** Zhao Li, Qinfeng Gao, Shuanglin Dong, Kang Dong, Yuling Xu, Yaoping Mei, Zhishuai Hou

**Affiliations:** 1 Key Laboratory of Mariculture (Ocean University of China), Ministry of Education (KLMME), Ocean University of China, Qingdao 266003, China; zhao-li@stu.ouc.edu.cn (Z.L.);; 2 Function Laboratory for Marine Fisheries Science and Food Production Processes, Qingdao National Laboratory for Marine Science and Technology, Qingdao 266100, China

**Keywords:** crowding stress, juvenile trout, seawater, HSL, glycerol

## Abstract

**Simple Summary:**

Crowding stress from stocking density is one of the critical stress factors in mariculture. Exposure of fish to chronic crowding stress substantially affects their well-being and physiological state, resulting in decreased growth performance, disordered metabolism, and consequent economic losses. In this research, we aimed to provide a deeper understanding of the effects of chronic crowding stress from stocking density on rainbow trout in mariculture. The obtained data offer unprecedented insights into the relationship between the growth performance and lipid metabolism of rainbow trout under chronic stress from stocking density in mariculture.

**Abstract:**

(1) Background: In aquaculture, chronic stress due to high stocking density impairs animals’ welfare and results in declined fishery production with low protein quality. However, most previous studies evaluated the effects of high stocking density on trout in freshwater rather than seawater. (2) Methods: Juvenile trout were reared for 84 days in circular tanks under three stocking densities, including low density (“LD”, 9.15 kg/m^3^), moderate density (“MD”, 13.65 kg/m^3^), and high density (“HD”, 27.31 kg/m^3^) in seawater. The final densities of LD, MD, and HD were 22.00, 32.05 and 52.24 kg/m^3^, respectively. Growth performance and lipid metabolism were evaluated. (3) Results: Growth performance and feeding efficiency were significantly reduced due to chronic stress under high density in mariculture. The digestive activity of lipids was promoted in the gut of HD fish, while the concentration of triglycerides was decreased in the blood. Furthermore, decreased acetyl-CoA carboxylase (ACC) and fatty acid synthase (FAS), increased hormone-sensitive lipase (HSL) concentrations, and activated hepatic β-oxidation processes were observed in trout under HD. Redundancy analysis showed that glycerol and HSL can be used as potential markers to evaluate the growth performance of trout in mariculture. (4) Conclusions: We showed that chronic high stocking density led to negative effects on growth performance, reduced de novo synthesis of fatty acids, and enhanced lipolysis.

## 1. Introduction

Aquaculture provides a large amount of high-quality protein in low-and middle-income countries [1,2]. In aquaculture, stocking density acts as a key matter of concern for the balance between maximum profit and minimum stressful responses [3,4]. Stressful responses trigger a cascade of biological events that enable organisms to resist death or reestablish physiological homeostasis, thus consuming the energy allocated for growth [5]. For example, previous studies showed that acute stress results in the dysregulation of physiological homeostasis and decreased growth in fish [6,7,8]. During stressful periods, the negative growth performance of fish is generally observed, while parameters related to growth rates are commonly considered indicators of stress and welfare [9,10]. Therefore, maintaining an optimum stocking density is important to maximize the profits of aquaculture and minimize fishery losses due to stressful responses.

On the one hand, due to the scarcity of freshwater, mariculture plays an increasingly important role in fishery production. Rainbow trout (*Oncorhychus mykiss*), an important euryhaline and economic species [11], is one of the Salmonidae species farmed in mariculture in recent years [12]. In mariculture, it is difficult to achieve intense aeration, oxygenation, and high-speed water flow. Moreover, part of the energy is consumed by osmotic regulation in a seawater environment, which reduces the ability of rainbow trout to cope with chronic stress [5]. On the other hand, optimum stocking densities are necessary to ensure animals’ welfare and maximize the benefits [13,14]. Stocking densities range from 2–80 kg/m^3^, depending on the oxygenation, water flow, and body size of the fish [14]. Excessively increasing the stocking density results in a decline in production due to stressful responses. Although previous studies showed that high stocking densities exert a negative impact on the growth performance of trout in freshwater [15,16,17], few have reported the effects of stocking density on rainbow trout in mariculture [18]. In mariculture, inappropriate stocking densities may result in stressful responses and further increase the energy expenditure of trout, thus leading to disordered energy for growth and survival. Therefore, it is vital to consider a reasonable stocking density of rainbow trout in mariculture.

Lipids are the predominant sources of metabolic energy and play essential roles in regulating growth, development, and reproduction [19]. Inappropriate stocking densities may disrupt the homeostasis of the lipid metabolism of cultured fish. In *Scophthalmus rhombus*, the triglycerides and total polar lipids were significantly increased under a high stocking density [20]. Moreover, to cope with the crowding stress, gilthead seabream (*Sparus aurata*) reared at a high stocking density decreased the hepatasomatic index and altered the composition of liver fatty acids [21]. These results suggest fish have species-specific regulation of the lipid metabolism in response to high stocking density. However, the regulatory mechanism of the lipid metabolism of trout under a high stocking density in seawater has not been intensively reported.

The intestines and liver play important roles in regulating lipid metabolism [22]. Nutrients are digested primarily through the intestine, and then the portal vein carries secondary bile acids and dietary metabolism from the intestines to the liver [23]. Previous studies showed that a high stocking density dysregulates the hepatic lipid metabolism and intestinal enzymes’ activities in largemouth bass (*Micropterus salmoides*) [24,25]. For fish in mariculture, the intestines are an important organ for maintaining osmotic pressure [26]. It can be maintained via consuming the energy provided by lipids, since lipids are the predominant sources of teleosts’ metabolic energy [19]. Considering that in mariculture, fish require more energy to cope with the stresses associated with high stocking density and osmoregulation, we hypothesized that high stocking densities might affect the growth and lipid metabolism of rainbow trout in mariculture.

In this study, we showed that a high stocking density in mariculture exerts negative effects on growth performance of rainbow trout via dysregulating the lipid metabolism. High stocking density enhanced hepatic lipolysis and activated the hepatic β-oxidation process of rainbow trout in mariculture, while reducing de novo synthesis of fatty acids. Our results provide a basis for understanding the alterations in the growth and lipid metabolism of rainbow trout in mariculture under chronic stress from stocking density.

## 2. Materials and Methods

### 2.1. Ethics Statement

This study was conducted in accordance with guidelines of Animal Research and Ethics Committee of Ocean University of China (permit number: 2014201) and the National Institutes of Health Guidelines for the Care and Use of Laboratory Animals (NIH publications No. 8023, revised 1987). No endangered or protected species was involved in this study, and the effect of gender was not considered because the juvenile trout were immature.

### 2.2. Experiment Design and Sample Collection

Rainbow trout (936 fish, 95.26 ± 8.33 g, 18.91 ± 0.49 cm) were obtained from a commercial trout farm in Rizhao, Shandong, China. Trout were acclimated in aerated fiberglass tanks (2.34 m diameter, 0.6 m depth, and 2.19 m^3^ water) with fresh water for one week, and the stocking density was ~15 kg/m^3^. After that, trout was transferred to salt water with a salinity of 10‰ for 7 days, and then the salinity of the culture media was gradually increased to 30‰ by adding deep seawater at a steady rate of 2‰ per day [27]. After a 2-week acclimation in salt water with a salinity of 30‰, the trout (100.17 ± 9.98 g, 18.99 ± 0.56 cm) were randomly distributed into nine polyethylene tanks (400 L, 1.0 m depth, 0.9 m diameter) with three different stocking densities, namely low density (LD), moderate density (MD), and high density (HD). During this experiment, each stocking density has three replications, and the trout were cultured for 84 days with a water temperature of 16.5 ± 0.5 °C, dissolved oxygen of around 7.3–9.0 mg/L, pH at 7.5 ± 0.1, less than 0.002 mg/L NH_3_, and a photoperiod of 12 h light and 12 h dark. The LD had an initial density at 9.15 kg/m^3^ (36 fish per tank, three replications), which is close to the stocking density of trout cultured in a small pond, while the MD had an initial density at 13.65 kg/m^3^ (54 fish per tank, three replications), which is close to the stocking density of trout cultured in raceways. The HD group had an initial density of 27.31 kg/m^3^ (108 fish per tank, three replications), which is close to the maximum stocking density of an aeration tank. The final densities of LD, MD, and HD were 22.00, 32.05, and 52.24 kg/m^3^, respectively.

Fish were fed twice a daily (at 08:00 and 17:00) at 1.5% biomass with commercial feed (Tianma group, China, 42% crude protein, 8% crude fat, 5% crude fiber, and 18% ash). Eighteen fish per treatment (six fish per tank) were weighed every 2 weeks to adjust the amount of feed according to the fishes’ weight and the water temperature (body weight and fork length are shown in Table 1 and Table A1). Fish were starved for 24 h before being euthanized, and nine individuals from each density group were anesthetized with a solution containing 100 mg/L of MS-222 at 4 different sampling times, including Day 0, Day 28, Day 56, and Day 84. The muscles, liver, serum, and gut were collected and stored at −80 °C until further analysis. The organs of three fish in each stocking density were randomly pooled as a sample for an analysis of the physiological and biochemical indicators. Each density group had three replicates.

### 2.3. Growth and Feeding Performance

After 14, 28, and 56 days, 18 fish (including the 9 sampling fish) were randomly selected per density group, and the body weight (BW) and fork length (FL) were recorded. All fish were measured at the end of the experiment for an evaluation of the growth and feeding. The growth and feeding performances were calculated as follows:

Fulton’s condition factor: CF (%) = (BW/FL^3^) × 100;

Feed conversion ratio: FCR (g/g) = (feed intake/(sampling BW − initial BW));

Weight gain rate: WG (%) = ((sampling BW − initial BW)/initial BW) × 100.

### 2.4. Organ Coefficients

After 28, 56, and 84 days, three fish were randomly selected per density group and the body weight (BW), the visceral mass (VM), liver (HM), and mesenteric adipose tissue (MM) were weighed. The organ coefficients were calculated as follows:

Visceral somatic indices: VSI (%) = VW/BW × 100;

Hepatopancreas somatic indices: HSI (%) = HM/BW × 100;

Mesenteric adipose somatic indices: MSI (%) = MM/BW × 100.

### 2.5. Muscle Composition

The dorsal muscles of 9 fishes in each density group were used for determination of the muscle composition via standard methods [28]. The moisture content was obtained by the dry weight of the samples at 105 °C for 24 h. The ash content was determined by burning the samples in heated furnaces at 550 °C for 6 h. The crude protein content was calculated with a Kjeldahl apparatus (nitrogen × 6.25). The crude fat content was determined from the lipids extracted with petroleum ether via a Soxhlet device (B-801, BUCHI Labortechnik AG, Flawil, Switzerland).

### 2.6. Measurement of Lipids’ Biochemical Indicators

A suite of key biochemical indicators of lipids was measured in the intestines, blood, and liver tissues from trout under different densities (*n* = 3). After homogenization using a tissue tearer in a normal PBS buffer (1×) on ice and centrifugation at 5000 rpm for 25 min at 4 °C, the supernatant of the intestine and liver pools was transferred to a new tube for biochemical assays. Blood was collected from the caudal vein of trout, and a pool of blood samples from three fish of the same group was considered as a replicate (*n* = 3 replicates per group). After centrifugation at 3500× *g* for 15 min at 4 °C, plasma was collected for the following biochemical measurements.

The concentrations of fatty acid synthase (FAS), hormone-sensitive lipase (HSL), acetyl-CoA carboxylase (ACC), L-carnitine, carnitine palmitoyltransferase I (CPT-1), ATP citrate lyase (ACLY), and acetyl coenzyme A (Ac-CoA) in the livers were determined according to the recommendations of commercially available ELISA kits that react specifically with fish (Jiancheng Bioengineering Institute, Nanjing, China). The concentrations of lipid metabolites, including TG, free fatty acids (FFA), glycerol, total bile acids (TBA), total cholesterol (T-CHO), low-density lipoprotein cholesterol (LDL-C), and high-density lipoprotein cholesterol (HDL-C) were determined in the intestine homogenate, liver homogenate, and plasma diluent using commercial colorimetric kits (Jiancheng Bioengineering Institute). The optical readings were obtained using a microplate reader (SpectraMax i3x, Molecular Devices, Silicon Valley, CA, USA).

### 2.7. Statistical Analyses

Results are presented as the mean ± standard deviation (SD). PCA was plotted by https://www.bioinformatics.com.cn (last accessed on 10 July 2023), an online platform for data analysis and visualization. Redundancy analysis (RDA) was performed using the OECloud tools at https://cloud.oebiotech.com/ (accessed on 2 April 2024). The Shapiro–Wilk test was used to check the normality of the data, and Levene’s test was used to check the homogeneity of the variances. One-way analysis of variance (ANOVA) followed by Duncan’s multiple range test (homogeneity) or Tamhane’s T2 multiple comparison test (inhomogeneity), with a significance level of *p* < 0.05, was used to evaluate the statistical differences in SPSS software version 26.0.

## 3. Results

### 3.1. Growth Performance

**Figure 1:**
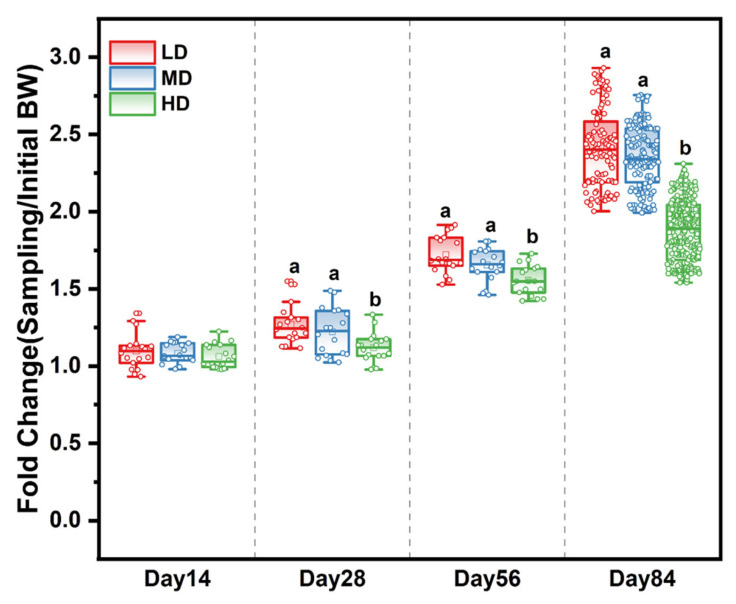
Fold change in body weight based on the initial body weight in different treatment groups after 14, 28, 56, and 84 days. Different stocking density groups with different letters are significantly different according to ANOVA models (*p* < 0.05).

The survival rates of the LD, MD, and HD groups were 93.52%, 94.44%, and 93.94% at the end of the experiment, respectively (Table A2). The effects of different stocking densities on the growth of trout are shown in Table 1. After 28, 56, and 84 days, those in the HD group had a significantly lower body weight and WG when compared with the LD and MD groups. Meanwhile, the fold change in body weight based on the initial body weight in the different treatment groups showed that stocking density affected the growth rate over time. The slowest growth rate was observed in the HD group (Figure 1), while the FCR of HD trout was significantly higher than that of LD and MD trout. Stocking density exerted no effects on fork length and condition factor from 0 to 56 days, while a significant difference was observed among the densities after 84 days (Table 1).

### 3.2. Organ Coefficients

**Figure 2:**
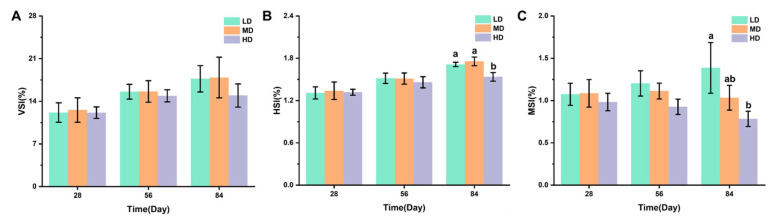
Changes in VSI (**A**), HSI (**B**), and MSI (**C**) values in different groups. Values represent the mean ± SD of three replicates (*n* = 3). Different stocking density groups with different letters are significantly different according to ANOVA models (*p* < 0.05).

Figure 2A–C shows the effects of different stocking densities on the organ coefficients of trout, including the VSI, HIS, and MSI values. After 28 and 56 days, no significant difference was observed in VSI, his, and MSI. After 84 days, no significant difference was observed in VSI under different stocking densities. The HD group had significantly lower HSI and MSI when compared with the LD group, while a significantly lower HSI was observed under HD when compared with MD.

### 3.3. Muscles’ Chemical Composition

The effects of different stocking densities on chemical composition of the muscles in trout are shown in Table 2. No significant difference was observed in crude protein and ash content under different stocking densities. The MD and HD groups had significantly lower moisture content and higher crude lipid content compared with LD.

### 3.4. Serum Lipid Metabolism

**Figure 3:**
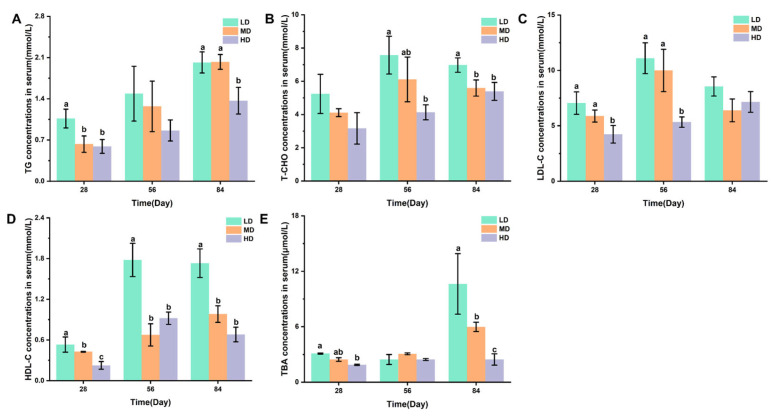
Changes in the serum lipid metabolism of rainbow trout stocked under different stocking densities. (**A**) TG, triglyceride; (**B**) T-CHO, total cholesterol; (**C**) LDL-C, low-density lipoprotein cholesterol; (**D**) HDL-C, high-density lipoprotein cholesterol; (**E**) TBA, total bile acids. Values represent the mean ± SD of three replicates (*n* = 3). Different stocking density groups with different letters are significantly different according to ANOVA models (*p* < 0.05).

Figure 3A,B shows the changes in TG and T-CHO concentrations in the serum of rainbow trout stocked under different stocking densities. Figure 3C–E shows the changes in the LDL-C, HDL-C, and TBA concentrations in the serum of rainbow trout stocked under different stocking densities. After 28 days, the HD group had significantly lower TG, LDL-C, HDL-C, and TBA contents when compared with the LD group. After 56 days, significantly lower T-CHO, LDL-C, and HDL-C contents were observed under HD when compared with LD. After 84 days, compared with the LD group, the HD group had significantly lower TG, T-CHO, HDL-C, and TBA contents.

### 3.5. Intestinal Lipid Metabolism

**Figure 4:**
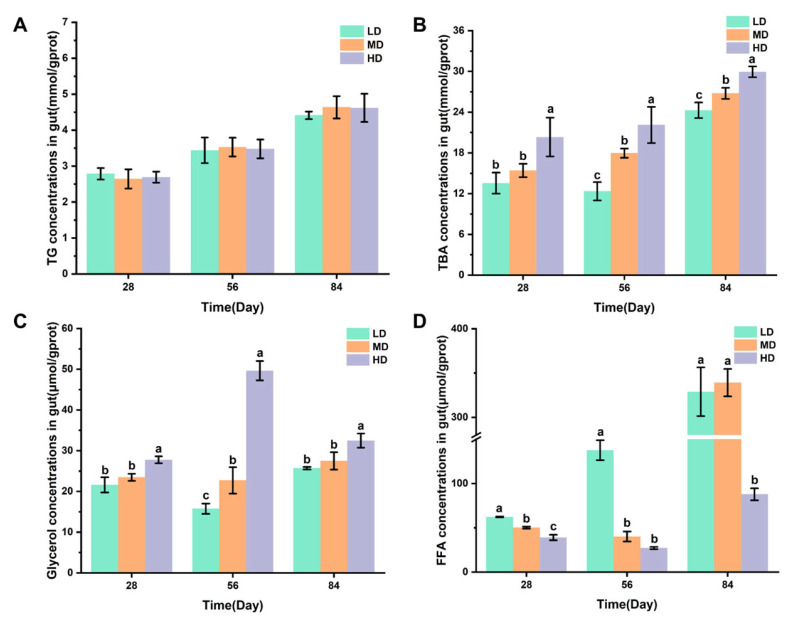
Changes in the gut lipid metabolism of rainbow trout stocked under different stocking densities. (**A**) TG, triglyceride; (**B**) TBA, total bile acids; (**C**) glycerol; (**D**) FFA, free fatty acids. Values represent the mean ± SD of three replicates (*n* = 3). Different stocking density groups with different letters are significantly different according to ANOVA models (*p* < 0.05).

Figure 4A,B shows the changes in the TG and TBA concentrations in intestines of rainbow trout stocked under different stocking densities. Figure 4C,D shows that the changes in glycerol and FFA concentrations in intestines of rainbow trout stocked under different stocking densities. No significant difference was observed in the TG content after 28, 56, and 84 days. The HD group had significantly higher TBA and glycerol contents after 28, 56, and 84 days when compared with the LD and MD groups. However, significantly lower FFA contents were observed in the HD group after 28, 56, and 84 days when compared with LD, while the HD group had significantly lower FFA contents after 28 and 84 days when compared with the MD group.

### 3.6. Liver Lipid Metabolism

**Figure 5:**
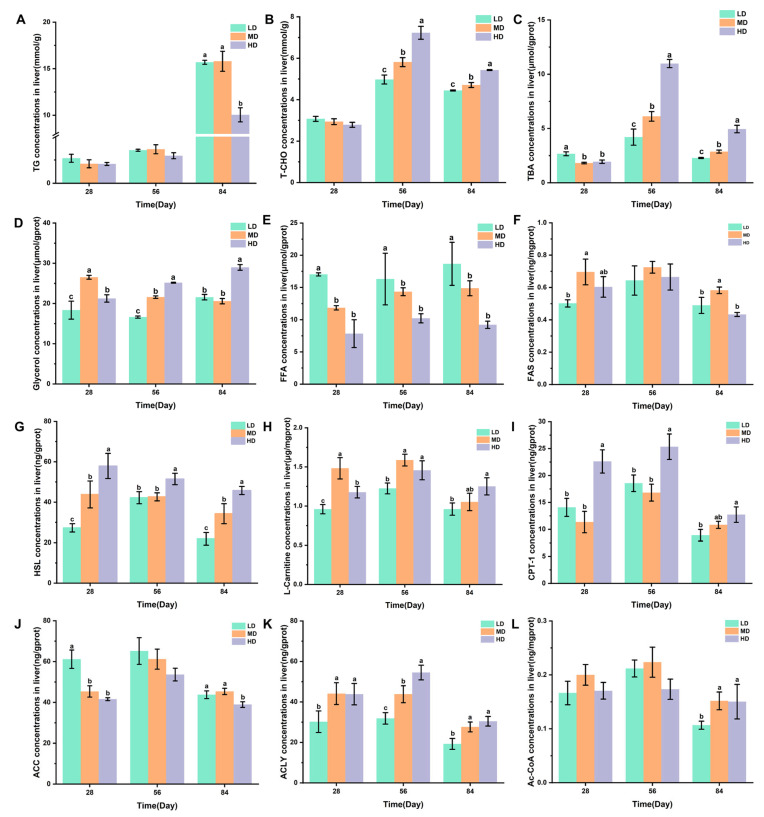
Changes in the liver lipid metabolism of rainbow trout stocked under different stocking densities. (**A**) TG, triglyceride; (**B**) T-CHO, total cholesterol; (**C**) TBA, total bile acids; (**D**) glycerol; (**E**) FFA, free fatty acids; (**F**) FAS, fatty acid synthase; (**G**) HSL, hormone-sensitive lipase; (**H**) L-carnitine; (**I**) CPT-1, carnitine palmitoyltransferase I; (**J**) ACC, acetyl-CoA carboxylase; (**K**) ACLY, ATP citrate lyase; (**L**) Ac-CoA, acetyl coenzyme A. Values represent the mean ± SD of three replicates (*n* = 3). Different stocking density groups with different letters are significantly different according to ANOVA models (*p* < 0.05).

Figure 5A–C shows the changes in the TG, T-CHO, and TBA concentrations in the liver of rainbow trout stocked under different stocking densities. The HD group had a significantly lower TG content after 84 days when compared with the LD and MD groups. Compared with the LD and MD groups, the HD group significantly higher T-CHO and TBA contents after 56 and 84 days, while the MD group had significantly higher T-CHO and TBA contents when compared with the LD group. The changes in the glycerol, FFA, and FAS concentrations in the livers of rainbow trout stocked under different stocking densities are shown in Figure 5D–F. HD produced significantly higher glycerol contents after 28, 56, and 84 days when compared with LD. Compared with MD and HD, LD produced significantly higher FFA contents after 28, 56, and 84 days. MD produced significantly higher FAS contents after 28 and 84 days. Figure 5G–L shows the changes in the HSL, L-carnitine, CPT-1, ACC, ACLY, and Ac-CoA concentrations in the livers of rainbow trout stocked under different stocking densities. HD produced significantly higher HSL, L-carnitine, CPT-1, and ACLY contents after 28, 56, and 84 days. However, HD produced significantly lower ACC contents after 28 and 84 days. Compared with MD and HD, LD produced significantly lower Ac-CoA contents after 84 days.

### 3.7. Principal Component Analyses (PCA) and Redundancy Analysis (RDA)

**Figure 6:**
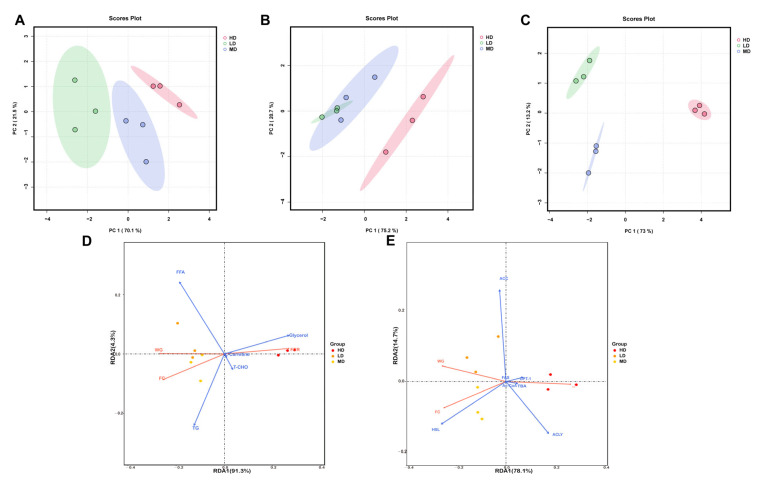
Multivariate analysis of biochemical lipid indicators in rainbow trout. Principal component analyses (PCA) of the biochemical lipid indicators of the intestines (**A**), serum (**B**), and liver (**C**) in rainbow trout. (**D**) Redundancy analysis (RDA) of growth performance and lipid substrate/metabolite variables. (**E**) Redundancy analysis (RDA) of growth performance and lipid–associated catalyzing enzyme variables.

The multivariate analysis of the biochemical lipid indicators of rainbow trout is shown in Figure 6. As can be seen from Figure 6A–C, three groups of samples can be clearly distinguished in the PCA plots. In the PCA results of three organs, the first axis captured 70.1%, 75.2%, and 73% of the total variability, while the second axis captured only 21.5%, 20.7%, and 13.2%. Meanwhile, the LD group always had the lowest coordinates on the first axis, while the HD group always had the highest coordinates.

The RDA plot revealed that glycerol concentration was positively correlated with FCR, but was negatively correlated with FC and WG (Figure 6D). However, in the lipid-associated catalyzing enzymes, the HSL concentration was positively correlated with WG and FC, but was negatively correlated with FCR (Figure 6E).

## 4. Discussion

Due to the scarcity of freshwater resources [2,29], freshwater trout culture might be constrained in the future. Mariculture plays an important role in alleviating pressure on freshwater resources. However, the mariculture environment is significantly different from the freshwater environment. Trout trigger the smoltification process to adapt to the high salinity of the mariculture environment, and smoltification results in significant alterations in the appearance, physiology, and behavior of trout [30,31]. Therefore, the energy homeostasis of growth, coping with stress, and osmoregulation is changed before (freshwater stage) and after (seawater stage) smoltification [32]. Inappropriate stocking densities lead to stress on trout, and most previous studies focused on the stocking densities of trout in freshwater rather than seawater environments. Therefore, we evaluated the optimal stocking density of trout in mariculture. We showed that chronic stress from high stocking density exerted negative effects on the growth performance of rainbow trout in mariculture by dysregulating the lipid metabolism.

**Figure A1:**
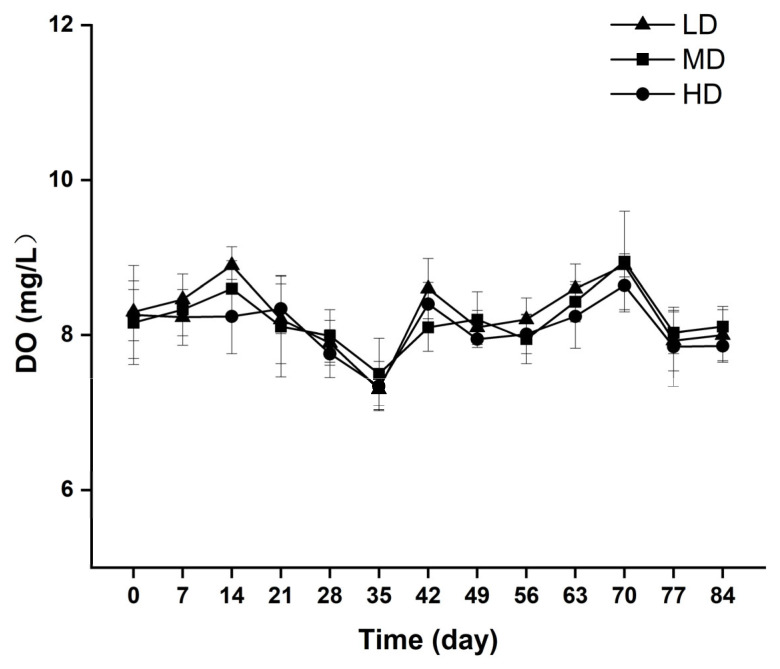
Dissolved oxygen (DO) under different stocking densities during the experiment. No significant differences were observed among three treatment groups at the same time point.

Chronic crowding stress is often accompanied by hypoxic stress, so we ensured adequate oxygenation of the rainbow trout by using flow-through aquaculture and aeration; the dissolved oxygen (DO) data are shown in Figure A1. We showed that chronic crowding stress exerted negative effects on the growth performance, potentially suggesting that a high stocking density results in reduced feeding efficiency and poor growth performance. Indeed, teleosts reared under a high stocking density require more food to cope with stressful responses [33]. The significantly increased FCR under the high stocking density suggested that a large amount of energy from food was consumed by coping with stress rather growth [34]. The high stocking density led to decreased HSI and MSI, suggesting that chronic stress dysregulated hepatic and intestinal physiology. CF is an important parameter associated with the general welfare of cultured fish, and we observed decreased CF due to the high stocking density.

**Figure 7:**
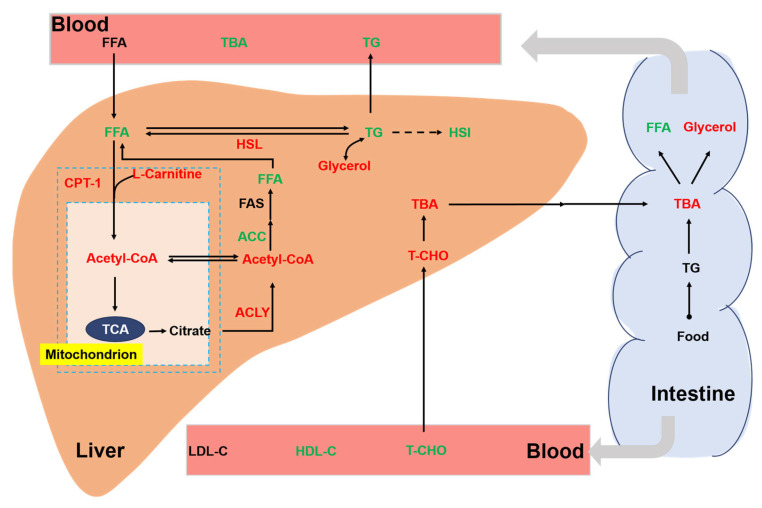
Integrative map of changes in the lipid metabolism of rainbow trout. Different colors represent significant upregulation or downregulation under HD when compared with LD. Red: upregulation; green: downregulation. TG, triglycerides; T-CHO, total cholesterol; TBA, total bile acids; glycerol; FFA, free fatty acids; FAS, fatty acid synthase; HSL, hormone-sensitive lipase; CPT-1, carnitine palmitoyltransferase I; ACC, acetyl-CoA carboxylase; ACLY, ATP citrate lyase; Ac-CoA, acetyl coenzyme A; LDL-C, low-density lipoprotein cholesterol; HDL-C, high-density lipoprotein cholesterol.

Chronic stress is an important environmental factor that modulates the lipid metabolism of aquatic animals. Previous studies have shown that chronic crowding stress increased the expression of fatty-acid-binding proteins related to lipid metabolism in fish [35]. In addition, chronic heat stress had profound effects on the lipid metabolism of fish by upregulating the related genes [36]. In our study, we also found a significant effect of chronic stress on the lipid metabolism of rainbow trout (Figure 7). In the liver, cholesterol is metabolized to bile acids. Bile acids can act as emulsifiers of lipids in the intestine and then be transported back to liver via bile ducts [37]. In this study, we showed that a high stocking density led to a significant increase in hepatic TBA. Consistently, the concentration of the T-CHO substrate used for the synthesis of bile acids was increased in the liver of trout under the high stocking density. Interestingly, a decreased T-CHO level was observed in the blood serum of rainbow trout under chronic stress, which agreed with a study of chronic stress in yellow catfish (*Pelteobagrus fulvidraco*) [38]. We also showed that chronic crowding stress significantly enhanced the hepatic expression of HSL. HSL regulates the rate-limiting step of the hydrolysis of TG into FFA and glycerol [39,40]. Upregulated HSL expression might indicate increased hydrolysis of TG and increased synthesis of FFA and glycerol. Consistently, we observed that trout under chronic crowding stress showed significantly decreased TG and increased glycerol concentrations. In addition, FAS is a key biosynthetic enzyme involved in the production of fatty acids from ACC [41]. The downregulated expression of FAS and ACC protein may decrease the synthesis of fatty acids, thus contributing to the lower concentrations of FFA. Consequently, chronic crowding stress triggered hepatic β-oxidation, which was characterized by the CPT-1 enzyme activity and L-carnitine content.

In our study, PCA showed that chronic crowding stress made a positive contribution to PC1, while the low stocking density made a negative contribution to PC1. These results suggested that chronic crowding stress led to significant changes in the biochemical lipid indicators of rainbow trout. Moreover, RDA was performed to assess the association between growth performance and biochemical lipid indicators in the liver. We showed that glycerol and HSL concentrations were strongly correlated with growth performance. The increased liver glycerol and HSL concentrations indicated the reduced growth performance of rainbow trout. Consistently, previous studies have found increased glycerol and HSL levels in teleosts under stress [42,43]. Therefore, hepatic glycerol and HSL can be used as markers to measure the growth performance of rainbow trout under stress.

## 5. Conclusions

The present results showed that chronic stress from high stocking density increased HSL concentrations, activated the hepatic β-oxidation process, and enhanced lipolysis, thus contributing to reduced crude lipid content and poor growth performance. Our study showed that glycerol and HSL in the liver can be used as markers to evaluate the growth performance of rainbow trout under stress. Our study could provide a basis for understanding the growth and lipid metabolism of rainbow trout under chronic stress from high stocking density in mariculture.

## Figures and Tables

**Table 1:** Growth performance of trout under different stocking densities. Rainbow trout were stocked under low (LD), moderate (MD), or high stocking densities (HD) for 84 days.

Time	Indices		Density Groups	
		LD	MD	HD
0–14 days	Body weight (g)	111.25 ± 11.67	109.18 ± 6.70	107.60 ± 7.72
	Fork length (cm)	19.86 ± 0.69	19.93 ± 0.62	19.84 ± 0.72
	Condition factor	1.42 ± 0.06	1.38 ± 0.07	1.38 ± 0.07
	FCR	2.05 ± 0.45	2.64 ± 0.91	3.17 ± 0.58
	WG (%)	10.90 ± 3.19	9.05 ± 3.19	7.70 ± 0.60
0–28 days	Body weight (g)	128.66 ± 12.82 ^a^	123.31 ± 15.45 ^a^	113.26 ± 9.71 ^b^
	Fork length (cm)	20.10 ± 0.78	19.95 ± 0.95	19.66 ± 0.70
	Condition factor	1.54 ± 0.11	1.54 ± 0.07	1.51 ± 0.07
	FCR	1.46 ± 0.14 ^b^	1.80 ± 0.0.24 ^b^	3.29 ± 0.40 ^a^
	WG (%)	28.25 ± 3.79 ^a^	23.18 ± 3.79 ^a^	13.35 ± 1.89 ^b^
0–56 days	Body weight (g)	174.43 ± 12.09 ^a^	166.92 ± 10.88 ^a^	157.54 ± 9.66 ^b^
	Fork length (cm)	21.61 ± 0.71	21.40 ± 0.65	21.23 ± 0.52
	Condition factor	1.73 ± 0.10	1.71 ± 0.12	1.71 ± 0.10
	FCR	1.50 ± 0.01 ^b^	1.60 ± 0.17 ^ab^	1.75 ± 0.05 ^a^
	WG (%)	73.85 ± 2.20 ^a^	66.72 ± 2.20 ^b^	57.68 ± 1.97 ^c^
0–84 days	Body weight (g)	244.40 ± 25.62 ^a^	237.44 ± 20.25 ^a^	189.95 ± 20.75 ^b^
	Fork length (cm)	23.70 ± 1.19 ^a^	23.30 ± 1.09 ^b^	22.04 ± 1.17 ^c^
	Condition factor	1.85 ± 0.26 ^b^	1.91 ± 0.28 ^a^	1.78 ± 0.20 ^c^
	FCR	1.41 ± 0.05 ^b^	1.38 ± 0.04 ^b^	1.95 ± 0.10 ^a^
	WG (%)	143.63 ± 3.25 ^a^	137.13 ± 3.25 ^b^	90.31 ± 3.03 ^c^
	Density (kg/m^3^)	22.00 ± 0.11	32.05 ± 0.29	51.34 ± 0.81

Different stocking density groups with different letters are significantly different according to ANOVA models (*p* < 0.05). FCR: feed conversion rate; WG: weight gain.

**Table 2:** Chemical compositions of the muscles of trout stocked at different stocking densities.

Item (%)	Density Groups		
	LD	MD	HD
Moisture	74.62 ± 0.36 ^b^	75.91 ± 0.50 ^a^	75.97 ± 0.20 ^a^
Crude protein	18.71 ± 0.76	18.63 ± 0.42	18.96 ± 0.38
Crude lipid	21.99 ± 1.89 ^a^	17.06 ± 2.66 ^b^	17.34 ± 0.78 ^b^
Ash	6.00 ± 0.85	5.68 ± 0.63	5.59 ± 1.06

Different stocking density groups with different letters are significantly different according to ANOVA models (*p* < 0.05).

## Data Availability

Data are contained within the article and Appendix A and Appendix B.

## Appendix A

**Table A1:** Increase in body weight and fork length excluding the time involved in Table 1.

Items	Group	Day 0	Day 42	Day 70
Body weight (g)	LD	101.32 ± 6.72	141.78 ± 8.19 ^a^	190.75 ± 11.31 ^a^
	MD	100.83 ± 6.27	135.89 ± 8.73 ^b^	182.58 ± 9.57 ^b^
	HD	101.24 ± 6.76	124.81 ± 7.80 ^c^	172.31 ± 13.47 ^c^
Fork length (cm)	LD	18.95 ± 0.53	20.15 ± 0.61	22.53 ± 0.56
	MD	19.04 ± 0.52	20.01 ± 0.66	22. 41 ± 0.62
	HD	18.97 ± 0.47	19.99 ± 0.57	21.98 ± 0.58

Different stocking density groups with different letters are significantly different according to ANOVA models (*p* < 0.05).

**Table A2:** The survival rate (%) of rainbow trout under different stocking densities at the end of the experiment.

Density Groups	LD	MD	HD	*p* Value
Survival rate (%)	93.52 ± 4.24	94.44 ± 1.85	93.94 ± 2.92	0.938

No significant difference among three groups according to ANOVA models (*p* < 0.05).
